# Neuronal Encoding of Texture in the Whisker Sensory Pathway

**DOI:** 10.1371/journal.pbio.0030017

**Published:** 2005-01-11

**Authors:** Ehsan Arabzadeh, Erik Zorzin, Mathew E Diamond

**Affiliations:** **1**Cognitive Neuroscience Sector, International School for Advanced StudiesTriesteItaly; Harvard University

## Abstract

A major challenge of sensory systems neuroscience is to quantify brain activity underlying perceptual experiences and to explain this activity as the outcome of elemental neuronal response properties. Rats make extremely fine discriminations of texture by “whisking” their vibrissae across an object's surface, yet the neuronal coding underlying texture sensations remains unknown. Measuring whisker vibrations during active whisking across surfaces, we found that each texture results in a unique “kinetic signature” defined by the temporal profile of whisker velocity. We presented these texture-induced vibrations as stimuli while recording responses of first-order sensory neurons and neurons in the whisker area of cerebral cortex. Each texture is encoded by a distinctive, temporally precise firing pattern. To look for the neuronal coding properties that give rise to texture-specific firing patterns, we delivered horizontal and vertical whisker movements that varied randomly in time (“white noise”) and found that the response probabilities of first-order neurons and cortical neurons vary systematically according to whisker speed and direction. We applied the velocity-tuned spike probabilities derived from white noise to the sequence of velocity features in the texture to construct a simulated texture response. The close match between the simulated and real responses indicates that texture coding originates in the selectivity of neurons to elemental kinetic events.

## Abstract 2

Rats move their whiskers rhythmically across surfaces to sense their environment. Textured surfaces induce distinct patterns of vibration in the whiskers that are encoded by central neural activity

## Introduction

One goal of sensory systems neuroscience is to understand how the representations of complex, natural stimuli arise from the basic response properties of neurons. The present experiments explore the representation of textures in the rat somatosensory system. Rats have texture discrimination capacities rivaling those of humans [1]. In rats, as in humans [2], object exploration in the tactile modality derives from active palpation. Thus, rats create sensory signals by sweeping their whiskers across surfaces in a rhythmic forward-backward cycle with a frequency ranging from 5 to 15 Hz [1,3,4,5]. Several hundred primary afferent fibers—“first-order neurons”—innervate specialized receptors on each whisker shaft [6], and these are excited by whisker movement. Signals travel along the sensory nerve, past the cell body in the trigeminal ganglion, to the brain stem. Here the first synapse is located. The axons of second-order neurons cross the brain midline and travel to the thalamic somatosensory nuclei, where the second synapse is located. Thalamic neurons project to the primary somatosensory cortex, conveying information to layer IV cell populations called “barrels” [7,8].

There have been no reports concerning the cortical or subcortical neuronal activity generated by whisking along irregular surfaces, and the differences in activity associated with two surfaces remain unknown [9]. However, recent work suggests the framework for a texture coding model. For nonnatural whisker deflections such as ramps [10], sinusoids [11,12], and temporally unstructured movement [13], first-order sensory neurons and cortical neurons emit spikes with probabilities that increase in proportion to stimulus velocity. This raises the possibility that neurons represent texture by encoding the kinetics of whisker vibrations. However, key elements of the model are untested. Does whisker movement across different textures produce distinct vibrations? If so, do neurons in the central pathway reliably report these vibrations? Through what coding mechanisms?

To answer these questions, the model must be challenged under conditions where the sensory input is precisely controlled and yet resembles what occurs during natural tactile behavior. Guided by this strategy, in anesthetized rats we produced whisker movements across textures while measuring vibrations of the whisker shaft. We then played back the identical vibrations to other rats, and measured the neuronal activity at two stages of the sensory pathway—the first-order neurons that innervate the sensory receptors, and the barrel cortex neurons, which are the first site of cortical integration. Texture discrimination depends on the integrity of the cortical barrels [14]. By measuring the activity of trigeminal ganglion neurons (the cell bodies of the first-order neurons), we investigated how the sweeping motion of whiskers along a surface is converted to a neuronal impulse code. By measuring activity in the cortex, we explored the neuronal representation that rats rely on to judge the identity of external objects [15,16]. Comparing two levels of the pathway, we reveal the transformation of neuronal signals at successive levels of integration, and show how the neuronal signals emerge from elemental feature extraction.

## Results

### Kinetic Signatures of Textures

**Figure 1:**
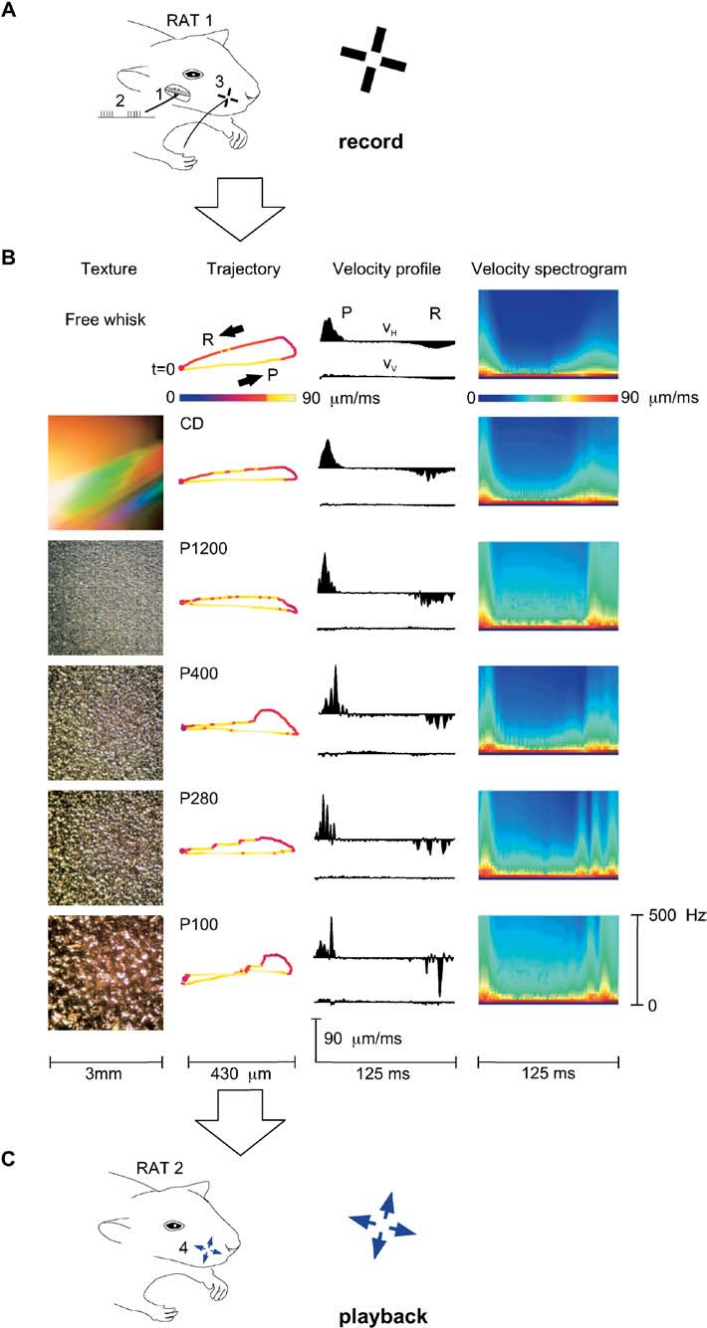
Collection and Playback of Texture Library (A) Whisker vibration data were collected during “electrical whisking,” induced by stimulation of the facial nerve (1) with pulse trains (2) in rat EW3. An optical sensor, shown schematically by two orthogonal light paths (3), monitored vertical and horizontal whisker motion of whisker C3. (B) “Texture” column: Photographs of the 5 surfaces used. “Trajectory” column: Sample whisker trajectories (first whisk of trial 50) associated with free whisking and the five surfaces. Each point, separated by 1 ms, gives the horizontal and vertical position; the trajectory begins with protraction (P) at *t* = 0 and terminates 125 ms later at the end of retraction (R). Speed is given by the color of each point. Note the irregularities—jumps, stops, and starts—induced by whisking on sandpaper. “Velocity profile” column: Whisker trajectories displayed according to the horizontal and vertical velocities (V_H_ and V_V,_ respectively). P refers to protraction phase (positive V_H_), and R to retraction phase (negative V_H_). In this and all figures, V_H_ and V_V_ were calculated 7,634 times per second. “Velocity spectrogram” column: Velocity spectrograms for each texture (see Materials and Methods). (C) Playback of the whisker trajectories to a second group of rats through a piezoelectric motor (4), shown schematically by the horizontal and vertical arrows at the base of the whisker.

The experimental strategy (Figure 1) was to collect records of the natural movement of whiskers across surfaces (Figure 1A) and use them as a stimulus set to probe the neuronal representation of texture (Figure 1C). In one group of rats (*n* = 3), we electrically stimulated cranial nerve VII, generating 8-Hz whisking movements [17,18] that resemble whisker trajectories in awake rats [4]. Meanwhile, whisker displacements transmitted to the receptors in the follicle were measured by an optical sensor placed 1 mm from the skin. The vertical and horizontal channels of the sensor (Video S1) reported whisker position with less than 3-μm spatial and 0.13-ms temporal resolution. Movements were measured under different conditions (Figure 1B, “texture” column): whisking with no object contact (“free whisk”), whisking on compact disk surface (smooth), and whisking on sandpapers of four different grades: P1200, P400, P280, P100 (from fine-grained to coarse-grained; Table 1). The surface was oriented so that the whisker rested on it and remained in contact during the entire whisk trajectory. The proximal edge of the surface was 7 mm from the base of the whisker. The illustrated data (Figure 1) come from whisker C3 in rat EW3.

**Table 1:**
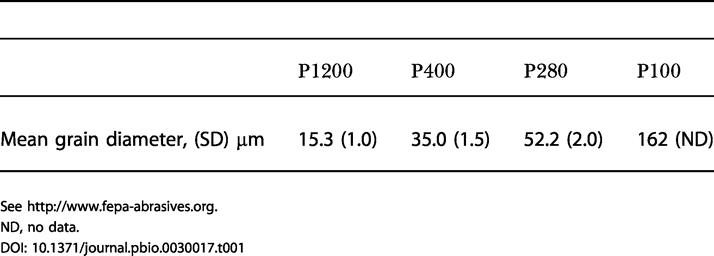
Parameters of the Sandpapers

See http://www.fepa-abrasives.org

ND, no data

Under free whisking conditions the trajectory was a smooth ellipsoid [4], the principal axis aligned with protraction and retraction movements (P and R, respectively, in Figure 1B, “trajectory” column). As the rat whisked on the compact disk surface, the trajectory was similar but covered a more restricted vertical range. In contrast, whisking across grainy surfaces produced irregularities in the trajectory, and each texture was associated with a characteristic whisker shaft vibration. These distinct “kinetic signatures” are evident in the velocity profile—that is, the temporal sequence of velocity features across the course of a whisk (Figure 1B, “velocity profile” column). Each velocity profile covers 125 ms (one complete forward and backward whisk) and consists of two histograms—horizontal (V_H_) and vertical (V_V_) velocity. For V_H_, whisker protraction (forward movement) is positive and whisker retraction (backward movement) negative. For V_V_, upward movement is positive and downward movement negative. To better visualize the time-varying frequency content of the velocity profiles, the velocity spectrograms are also plotted (Figure 1B, “velocity spectrogram” column). The spectrograms were formed by computing the magnitude of all sinusoidal components (0–500 Hz) of the velocity profile in a 6-ms wide window, and then sliding the window with 0.13-ms time steps (see Materials and Methods). The velocity profile and the velocity spectrogram, taken together, illustrate the kinetic features that make each texture unique—the duration and frequency content of each velocity peak, as well as the number of peaks and the temporal spacing between them.

In a different group of rats, the texture-induced vibrations were played back to the base of a whisker (Figure 1C and Video S2) and neuronal activity was recorded. Construction of the stimulus sequence off-line allowed us to smoothly “stitch together” the vibrations: The transitions between textures and free whisks were always inserted at time zero, corresponding to the point of maximal retraction, when whisker velocity was zero. The replay was an accurate replica of the motion recorded during electrical whisking (Figure S1).

### Receptor and Cortical Coding Properties

**Figure 2:**
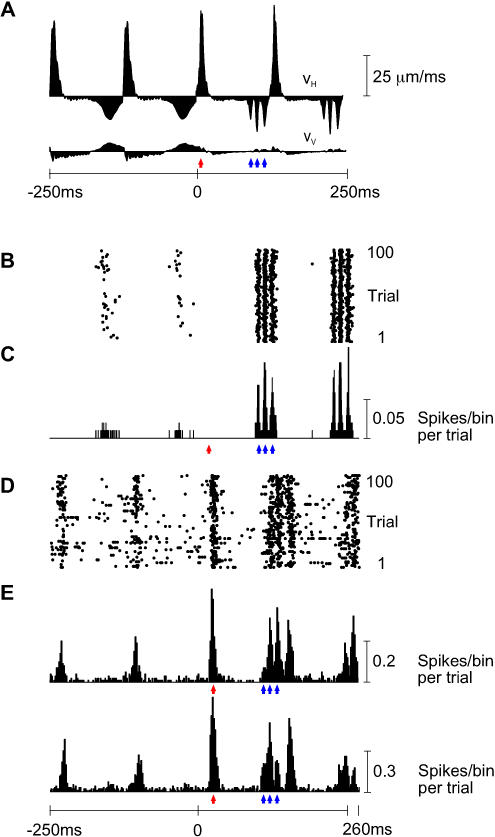
Sensory Receptor and Cortical Coding Properties (A) V_H_ and V_V_ for two free whisks followed by two P280 whisks. The labeling conventions are as in Figure 1B. Each presented trial was unique due to small variations in whisker trajectory even on the same surface (Figure 7); the illustrated velocity profiles are the averages of 100 trials. The red arrowhead indicates the time of the first V_H_ peak during whisker protraction on P280; blue arrowheads indicate the times of the three V_H_ peaks during whisker retraction on P280. (B) Raster plot of first-order neuron aligned with the whisker trajectories, in response to 100 unique trials. Stimuli were applied to whisker E4. (C) PSTH of first-order neuron with 0.2-ms bins. Blue arrowheads indicate the times of maximum response to the three peaks in retraction velocity. The red arrowhead indicates the expected time of response to the peak in protraction velocity; however, the neuron did not respond to whisker protraction. (D) Raster plot for the cortical neuron cluster recorded simultaneously with the first-order neuron. (E) Two cortical PSTHs, both with 2-ms bins. The upper PSTH corresponds to the raster plot in (D); the lower PSTH is from a second cortical neuron cluster recorded simultaneously at a neighboring electrode (distance 560 μm). Blue and red arrowheads indicate the times of maximum response to the peaks in whisker protraction and retraction velocity, carried down from (A). The cortical neuron clusters responded to high velocities for both protraction and retraction. Because the first two peaks in retraction velocity were separated by just 7 ms, the resulting peaks in cortical response were fused. All PSTHs are extended to 260 ms to show responses to the final velocity feature.

The physiological dataset (seven rats) consists of six first-order neuron recordings, five cortical cluster recordings, and seven “paired” recordings—simultaneous first-order neuron and cortical cluster. The principal result is that time-varying neuronal activity in the trigeminal ganglion and cortex captured the kinetic features of the texture-induced vibrations. From the same texture library given in Figure 1 (rat EW3, whisker C3), Figure 2A gives the velocity profile, averaged across 100 trials, for two free whisks (−250 to 0 ms) followed by two whisks on P280 sandpaper (0 to 250 ms). Note the distinct kinetic signatures of whisker movement: Unlike free whisking, the coarse (P280) sandpaper caused irregular bursts of high and low velocity, particularly during whisker retraction. The response of one first-order neuron (named Zurvan) is shown in Figure 2B as a raster plot of 100 trials and in Figure 2C as a peristimulus time histogram (PSTH). Several coding properties are evident: (i) The first-order neuron fired a greater number of spikes for the coarse texture than for free whisks; (ii) spikes were closely aligned to instants in which the whisker moved at high velocity (blue arrowheads); (iii) it fired in a reproducible manner across trials—the spikes were aligned; and (iv) it was selective to whisker retraction (did not fire for high-velocity protractions; red arrowhead).

Two barrel cortex neuron clusters were recorded simultaneously with the first-order neuron, allowing direct comparison of different stations along the sensory pathway. Figure 2D shows the raster plot for one of the cortical clusters, while Figure 2E shows PSTHs for both cortical clusters. Like the first-order neuron, the cortical clusters responded to high velocities (arrowheads, Figure 2A, 2C, and 2E) and, as a result, fired a greater number of spikes for P280 sandpaper than for free whisks. Key differences from the first-order neuron are clear: (i) The cortical neuron clusters fired in a less reproducible manner across trials—there was more variability in the number of spikes per whisk and in the temporal alignment of spikes; and (ii) they fired for both whisker protraction and retraction (red and blue arrowheads, respectively).

**Figure 3:**
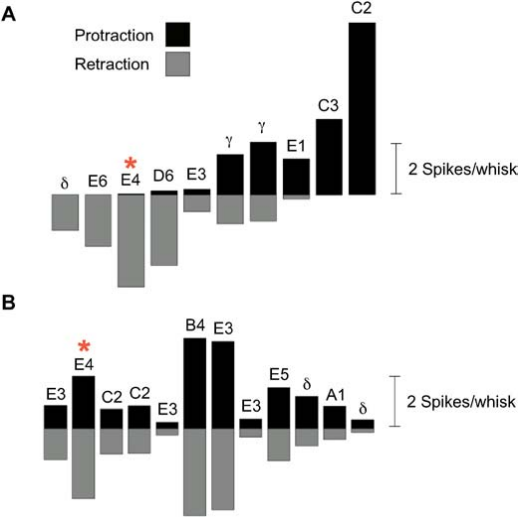
Directional Selectivity in First-Order and Cortical Neurons (A) Mean spike count per whisk for ten first-order neurons separated into protraction and retraction phases. Responses to free-whisk and all textures were combined, giving a total of 8,000 whisks. First-order neurons are arranged from left to right according to their retraction:protraction spike count ratio. Five first-order neurons preferred retraction, three preferred retraction, and two responded to both phases. Principal whisker of each neuron is indicated. The neuron Zurvan is indicated by an asterisk. (B) Same analysis for 12 cortical clusters. Individual cortical neuron clusters did not present a clear preference for either retraction or protraction. Conclusions about single unit directional selectivity cannot be drawn, however, because the directional selectivity of any cluster must always be less than that of the most selective single unit in the cluster. The neuron cluster (Figure 2D, 2E) recorded simultaneously with Zurvan is indicated by an asterisk.

The selectivity of first-order neurons and cortical clusters for the direction of whisker movement is described in more detail in Figure 3.

### Texture Coding by Firing Rate

**Figure 4:**
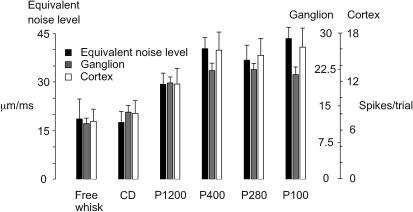
Texture Coding by Firing Rate Equivalent noise level (plus SD) of texture-induced vibrations averaged across 100 trials of 500 ms each (see Materials and Methods). Average spike count per trial (plus SD) pooled from ten first-order neurons and 12 cortical neuron clusters. Note separate scales for spike counts of neurons recorded in Ganglion and Cortex.

To permit sensory discriminations, some properties of neuronal firing must vary systematically from texture to texture. Earlier work [11,12] showed that neuronal firing rate, in response to sinusoidal whisker movement, is dictated by mean vibration speed, proportional to the product of amplitude and frequency, *Xω* (referred to in previous publications as *Af*). The generalization of *Xω* to the natural, texture-induced vibration is 〈|*X(ω,τ)*|*ω*〉_Ω,Τ_, a quantity known as “equivalent noise level” (see Materials and Methods). In Figure 4, we compare response magnitude for the full population of first-order and cortical neurons to each texture's equivalent noise level. The Pearson correlation coefficient between equivalent noise level and spike count was 0.93 for the first-order neurons and 0.99 for the cortex. This finding indicates that neuronal spike count is a function of the magnitude of the composite frequency components of the whisker vibration, whether the stimulus is a simple sinusoid (where all the power is at a single frequency) or a complex, texture-induced vibration.

### Texture Coding by Firing Patterns

**Figure 5:**
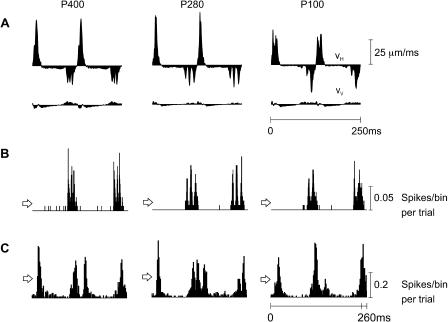
Texture Coding by Firing Patterns (A) V_H_ and V_V_ for two whisks on texture P400 (left), P280 (middle), and P100 (right). Each illustrated velocity profile is the average of 100 unique profiles. (B) First-order neuron PSTHs (0.2-ms bins) aligned with the whisker trajectories. (C) Cortical PSTHs (2-ms bins). PSTHs are extended to 260 ms. The arrowheads on the left side of PSTHs indicate mean firing rates.

**Figure 6:**
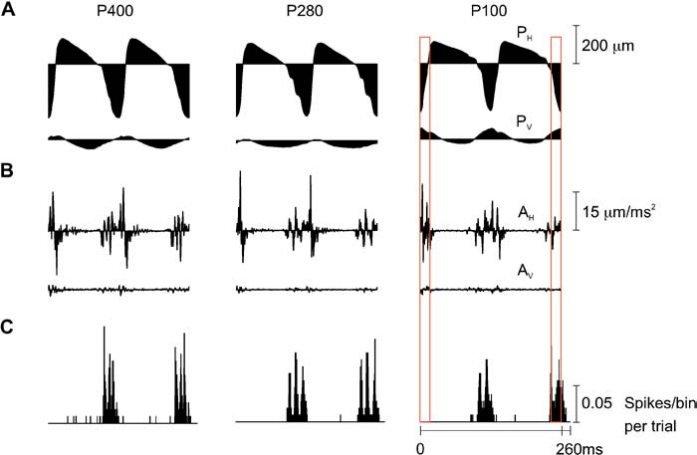
Test for First-Order Neuron Encoding of Position and Acceleration To investigate whether first-order neurons represented stimulus features other than velocity, we repeated the same analysis as in Figure 5, in relation to whisker position (A) and acceleration (B), because it has been suggested that neuronal activity is determined by these stimulus parameters [10,13]. Alignment between the PSTH (C) and stimulus position or acceleration revealed no consistent correlation. For texture P100, the boxes extending across A, B, and C highlight the absence of correlation. For example, two periods with similar positions produced first no spikes (red-outlined box on left) and then a large response (red-outlined box on right). Moreover, high acceleration (left box) produced no spikes, while lower levels of acceleration (right box) produced a large response. For this neuron, only velocity was encoded.

Since different sandpapers can induce vibrations with similar equivalent noise levels and thereby evoke similar spike counts (e.g., P400, P280, and P100 in Figure 4), we must expect additional texture coding mechanisms to be at work. We therefore examined more closely the neurons shown in Figure 2, on the hypothesis that spike patterns might carry texture-specific information. V_H_ and V_V_ profiles associated with two whisks on each texture are plotted (Figure 5A), together with PSTHs for the first-order neuron (Figure 5B) and the cortical neuron cluster (Figure 5C). Textures evoking similar spike counts due to similar equivalent noise levels were readily distinguished by spike patterns. The patterns arose from the alignment of spikes to the velocity profile of the input vibration. An assessment of spike alignment to other stimulus features (whisker position and whisker acceleration) is given in Figure 6 and indicates that these features were reported less reliably than whisker velocity. The first-order neuron reported the velocity profile for whisker retraction, while the cortical neuron cluster reported both protraction and retraction profiles, albeit with lower fidelity to individual velocity features.

### Sources of Neuronal Variability

**Figure 7:**
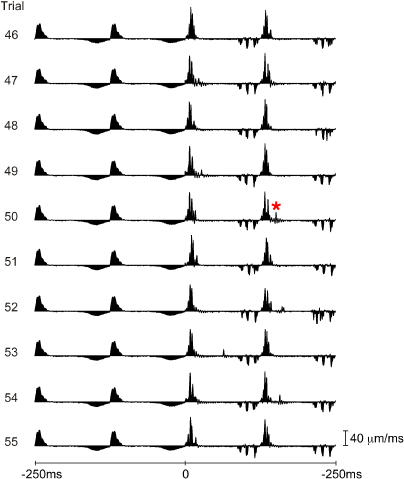
Velocity Profile Variability across Trials Ten successive trials are shown (numbers 46–55), each trial composed of the final two free-whisks (–250 to 0 ms) and the first two whisks on P280 (0 to 250 ms). Free whisk velocity profiles varied little across trials. When the whisker swept across P280 repeatedly, the fundamental kinetic signature was conserved (e.g., the three peaks in retraction velocity for P280) but minute details of the profile varied—note, for example, the velocity event (red asterisk) that occurred uniquely on trial 50.

In a number of sensory modalities, first-order neuron responses can be remarkably reliable when a stimulus is presented repeatedly [19], whereas cortical responses vary across trials [20]. It is of interest to elucidate the mechanisms that permit reliable first-order neuron responses and, by the same token, to identify the sources of trial-to-trial variability among cortical neurons. In the data shown so far, the 100 trials for a given texture were composed of 400 unique whisks (four whisks per trial). Each whisk differed in the minute details of its trajectory (Figure 7). To discover the origin of neuronal variability, we selected trial 50 for each texture and repeated the four-whisk sequence 100 times. If neuronal variability originates purely in stimulus variability, it will disappear across repeated trials; variability due to internal brain fluctuations, however, will remain.

**Figure 8:**
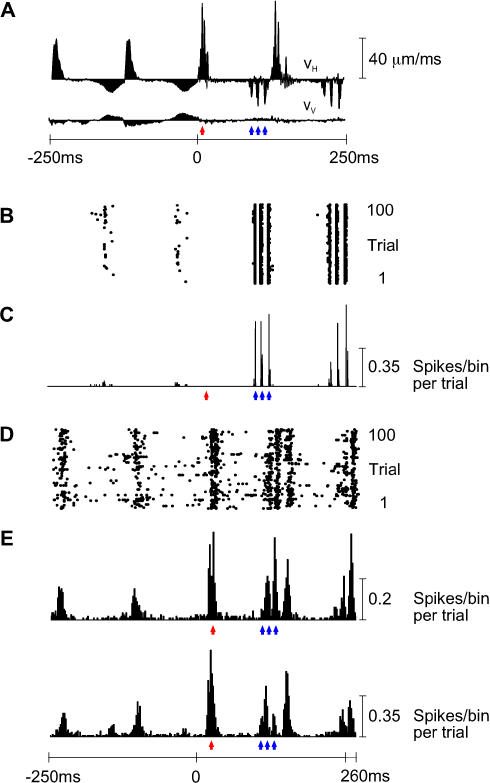
Sources of Neuronal Variability (A) V_H_ and V_V_ across the final two free whisks and the first two P280 whisks of trial number 50. Here, as in Figure 2, the red arrowhead indicates peak whisker velocity during protraction, and blue arrowheads indicate the peak whisker velocities during retraction. (B and C) First-order neuron raster plot (B) and PSTH (C), aligned with the whisker trajectories, for 100 stimulus repetitions. Due to the temporal precision of neuronal responses, the vertical scale of the PSTH has been altered (compare to Figures 2 and 5) to reflect the large numbers of spikes within single bins. (D) Cortical neuron cluster raster plot. (E) Two cortical PSTHs from activity recorded simultaneously with the first-order neuron. The upper PSTH corresponds to the raster plot in (D); the lower PSTH is derived from a second cortical neuron cluster recorded simultaneously at a neighboring electrode (distance of 560 μm). PSTHs have 0.2-ms bins for the first-order neuron and 2-ms bins for the cortical neuron clusters. All PSTHs are extended to 260 ms to show responses to the final velocity feature. Response peaks are signaled by red and blue arrowheads according to the velocity events that evoked them.

The velocity profile for the final two free whisks (−250 to 0 ms) and the first two P280 whisks (0 to 250 ms) of trial 50 is given in Figure 8A. The response of the first-order neuron is shown as a raster plot (Figure 8B) and a PSTH (Figure 8C). These can be compared to responses of the same cell in Figure 2B and 2C. Response was nearly identical on each trial, because of the precise temporal alignment of spikes on the high-velocity events. Indeed, some stimulus features evoked 0.7–0.8 spikes per bin per trial, meaning that neuronal jitter fell within the 0.2-ms PSTH bin size.

In Figure 8D and 8E, the cortical response to repeated trials is presented. Direct, quantitative comparisons between the variability of first-order responses and that of cortical responses cannot be made, because the cortical recordings were made from multi-neuron clusters. However, the cortical response to repeated trials can be compared to the same cluster's response to 100 unique trials in Figure 2D and 2E. Eliminating trial-to-trial variability in the timing of stimulus features reduced but did not eliminate neuronal jitter.

**Figure 9:**
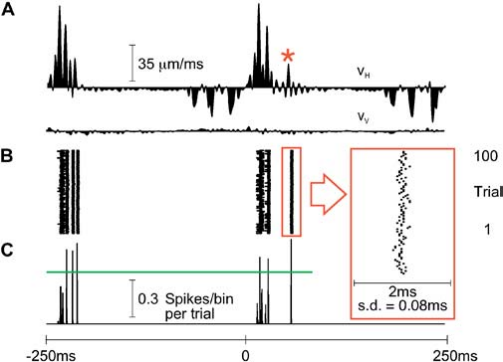
Precision of a First-Order Neuron (A) V_H_ and V_V_ across the first two P280 whisks of trial number 50 (see Figure 7). (B and C) Raster plot (B) and PSTH (C) of the first-order neuron for 100 repetitions of the stimulus given in (A). Inset in red frame shows a magnified view of spikes emitted in response to a single velocity event (red asterisk in [A]) and their SD in time. The same measurement of jitter was carried out for each of the response peaks that surpassed the green horizontal line (see text).

The remarkable response locking of first-order neurons to stimulus features is further highlighted in Figure 9. Unlike Zurvan, the illustrated neuron was selective to whisker protraction. For texture P280, the velocity histograms (Figure 9A) contained well-separated peaks during protraction. With 100 repetitions of trial number 50, the raster plot (Figure 9B) and PSTH (Figure 9C) yielded discrete response peaks aligned with high-velocity protraction events. Applying the green horizontal line to the PSTH as a threshold, we extracted six separable response clusters. Every cluster contained exactly 100 spikes resulting from one spike per trial for each velocity event. The final response was evoked by a clear protraction event (red asterisk in Figure 9A) and was selected for closer inspection (red inset). Here, the 100 spikes spanned a range of 0.38 ms; standard deviation (SD) in spike time was 0.08 ms. For the five preceding response clusters, spike time SDs were 0.13, 0.09, 0.09, 0.13, and 0.11 ms. The minimum measured spike time SD in the dataset was 0.07 ms. These must be taken as underestimates of spike time precision, given that they include measurement noise inherent to the recording system (e.g., the SD in spike time caused by digitizing the action potential threshold crossing time at 30 samples per ms is nearly 0.02 ms).

From these observations we conclude that, under our experimental conditions, the trial-to-trial response variability of first-order neurons is caused exclusively by stimulus jitter, whereas that of cortical neurons results mainly from variations across time in sensory integration, and must emerge at some integration site between the trigeminal ganglion and cortex. A question of current interest is whether the variability in cortical responses results from noise and imprecision in neuronal integration [20], or else reflects functionally significant modulations in responsiveness [21,22,23].

### From Response Properties to Natural Responses

We hypothesize that the firing patterns of first-order and cortical neurons during presentation of textures can be explained by their extraction of elemental features from the complex input signal, and that these elemental features are bursts of high velocity. To test this directly, we presented a “white noise” stimulus in which the two stimulus features V_H_ and V_V_ varied randomly across time. Responses to noise stimuli allowed us to quantify velocity sensitivity and then to generate simulated spike trains based on the sequence of velocity events in the actual texture-induced vibrations. Finally, comparison between simulated and observed responses reveals the extent to which the responses to natural stimuli are explained by neuronal selectivity to velocity features: If simulated responses closely resemble real responses, we can conclude that neurons are in fact operating on natural texture stimuli according to their tuning to elemental kinetic events.

**Figure 10:**
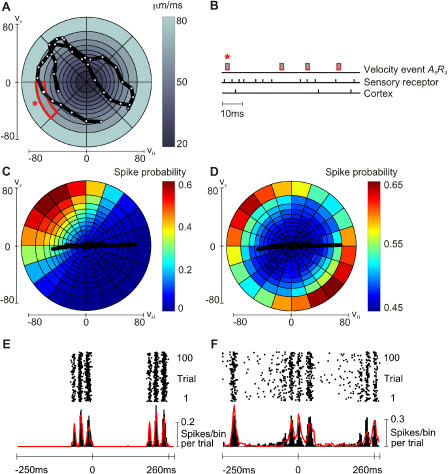
Velocity Tuning Curves and Simulated Texture Responses (A) A 5-ms trajectory of velocity white noise. Radial coordinates give V_H_, V_V_. Velocity space was subdivided such that each segment included the same number of events (3,435,300). One segment (red outline) is selected for further explanation (see text). (B) 100-ms ganglion and cortical spike train aligned below occurrences of the velocity event of interest (red bar). After each such event, spike times were accumulated to build up a spike probability profile. (C) First-order neuron spike probabilities, given by color scale, in relation to joint *A,R* events. To estimate the tuning curve in finer detail, the number of angles was increased to 20. Each segment now contains about 1,374,120 velocity events. One P280 whisk trajectory is superimposed. (D) Spike probabilities for cortical neuron cluster, given by color scale, in relation to joint *A,R* events. One P280 whisk trajectory is superimposed. (E) Simulated raster plot for first-order neuron and simulated (black) and real (red) PSTHs. (F) Simulated raster plot for cortical neuron cluster and simulated (black) and real (red) PSTHs.

Typically, neuronal tuning curves are mapped out using an “unbiased” stimulus—a stimulus that avoids the temporal correlations present in natural stimuli. The first step, therefore, was to map out how first-order and cortical neurons encode whisker kinetic features when these features are extracted from the context of the natural stimulus. We applied a stimulus that varied randomly in velocity—Gaussian velocity noise—and therefore was not constrained by the velocity patterns present in texture trajectories (see Materials and Methods). Figure 10A tracks V_H_ and V_V_ (white circles) across 5 ms of filtered white noise.

We then constructed firing probability profiles in relation to millions of occurrences of each velocity event, such as the velocity event in the fifth angular sector and ninth radial sector, or *A_5_,R_9_* (red outline in Figure 10A). Figure 10B shows how response probabilities were constructed in relation to this particular velocity event. The first trace shows the occurrences of *A_5_,R_9_* across a 100-ms window—the first occurrence of the event (asterisk in Figure 10A) corresponds to the crossing of *A_5_,R_9_* in Figure 10A (also marked by an asterisk). Below, first-order sensory neuron and cortical spike times are shown. After 10 min of stimulus noise, the postevent spike probability profiles associated with *A_5_,R_9_* and all other events could be constructed.

For the first-order neuron Zurvan, spike probabilities in the 1–2-ms postevent interval are given in Figure 10C for all joint events (*A,R*). To construct the neuron's “tuning curve” in finer detail, velocity space was subdivided into 20 angular and 10 radial segments. The neuron emitted spikes with increasing probability as velocity increased, but only for restricted directions, preferring high speeds that combined retraction (negative horizontal velocity) and upward movement (positive vertical velocity). For the simultaneously recorded cortical neuron cluster, spike probabilities in the 5–20-ms poststimulus interval are given in Figure 10D; like the first-order neuron, the cortical cluster emitted spikes with increasing probability as speed increased, but its directional selectivity was less pronounced and was radially symmetric.

Can the neurons' responses to complex, natural stimuli be explained as the outcome of these elemental tuning properties? To find out, we projected whisk velocity trajectories upon the white noise-derived tuning curves. One whisk (first whisk of trial 50) on P280 sandpaper is depicted on both tuning curves. The first observation is that the intersection of the velocity trajectory with the tuning curves explains why the first-order neuron was selective for whisker retraction while the cortical cluster was directionally nonselective (see Figures 2 and 5).

Figures 10E and 10F show the simulated responses of the first-order neuron and cortical cluster to two P280 whisks, delivered 100 times. The simulation was of 100 unique trials (see Figure 2) rather than repeated trials (see Figure 8). Thus, on each trial the minute details of the whisks gave rise to a unique sequence of *P*(*t*) and a corresponding raster plot for that trial. The 100-trial raster for one run of the simulation was summated to form a PSTH. The close match between the simulated PSTHs (black) and the real PSTHs (red lines, reproduced from Figure 2C and 2E) indicates that the real responses to natural stimuli could be explained by neuronal selectivity to velocity features. The Pearson correlation coefficients between the predicted and observed PSTHs were 0.94 for the first-order neuron and 0.84 for the cortical cluster. Because these correlation values fall within the range obtained by comparing two real PSTHs generated from separate sets of 50 trials, we conclude that simulated spike trains are as similar to real spike trains as real spike trains are to each other. Thus, the velocity feature extraction properties of the neurons are sufficient to explain texture responses.

## Discussion

Texture coding appears to derive from two fundamental processes: First, the whisker transmits a “kinetic signature” of the palpated surface to the receptors in the follicle. Second, the first-order neurons relay to the whisker region of cortex (through intervening stations) precise information about the kinetic features transmitted to the follicle. One kinetic feature is the “equivalent noise level”: Spike counts per whisk both for first-order neurons and for cortical neurons are proportional to the equivalent noise level of the texture-induced vibration. Thus, when texture vibrations differ in this quantity, neuronal spike counts also differ and thereby carry information that could, by itself, separate the textures. By the same token, when texture vibrations have similar equivalent noise levels, spike counts per whisk appear not to carry sufficient information. The second kinetic feature, then, is the temporal sequence of velocity events—distinctive velocity profiles induce distinctive temporal patterns in the spike trains with spike alignment of better than 0.2 ms in the first-order neurons and a few ms in the cortex.

The stimulus playback method used here might not produce the identical input to the sensory receptor as occurs during active whisking [18]. The optical sensor at the base of the whisker did not register the bending nor tension (pulling) of the whisker. Moreover, active muscle contractions might affect sensory receptors at the interface between the whisker shaft and the inner membrane of the follicle. Thus, additional information related to surface texture might be available to the sensory system. In the present dataset, even after the possible loss of some texture-dependent information due to passive stimulation, the neuronal responses afforded a high degree of discriminability. We interpret the dataset as showing that vibration patterns, by themselves, must be a fundamental feature supporting the neuronal coding for texture. This awaits confirmation in experiments in actively whisking rats.

A classical approach to investigating sensory coding is to map the relationship between well-controlled artificial sensory stimuli and evoked neuronal activity. This can provide a complete description of neuronal feature extraction properties [24,25], but it sheds little light on the brain activity underlying normal perceptual experiences. Moreover, the processing mechanisms that have evolved to extract behaviorally relevant information may operate inefficiently during artificial stimulation [26]. Another approach [27,28] is to measure neuronal activity during natural stimuli (i.e., visual scenes or animal calls). Here, the drawback is that the features evoking spikes during natural stimulation can be multidimensional, complex, and difficult to quantify, offering only limited insight into sensory processing mechanisms [27].

In principle, one can bridge the gap between artificial and natural stimuli by (i) measuring neuronal activity during ecologically relevant stimuli, stimuli that are collected from an animal's normal interaction with the environment, (ii) constructing tuning curves under artificial stimulation (usually white noise), and (iii) applying the tuning curves to the natural stimuli to test whether they account for the observed response. Because artificial stimuli only partially cover dimensions of stimulus space present in natural stimuli, and because of neuronal nonlinearity, this procedure typically provides neuronal simulations that match real neuronal output with a correlation of less than 0.5 [27,29,30]. Yet, the present experiments followed this same procedure and generated simulated PSTHs that were highly correlated with real responses (first-order neuron, 0.94; cortex, 0.84), approaching the upper bound set by the trial-to-trial variability that limits the correlation even between two real PSTHs. The simulations were successful for two reasons. First, recent work [11,12] uncovered the critical physical dimension—velocity—encoded by neurons. Second, receptor and cortical stimulus integration is linear to a first approximation—ongoing responses depend upon an integration process where preceding events affect the neurons independently of one another.

As an alternative to the temporal model outlined here, a spatial model for texture coding has recently been proposed. It begins from the observation that whisker length varies systematically across the anterior-posterior dimension of the rat's snout [31]. When stimulated near the distal end, the posterior whiskers resonate at lower frequencies than do the shorter, anterior whiskers [32]. If different textures cause systematically different vibration frequencies, there might be texture-specific differences in the magnitude of vibration of posterior versus anterior whiskers [33]. Because whisker position is relayed in a somatotopic manner to the cerebral cortex [7], texture could be encoded by the location of the focus of activity in barrel cortex, much like frequency is encoded by the tonotopic organization of the cochlea and the auditory cortex.

The resonance frequency hypothesis predicts that rats would fail to discriminate between surfaces using just a single whisker, yet they have been shown to perform texture discriminations after progressive clippings down to one or two whiskers [34]. Our results help explain the behavioral findings by emphasizing that, although additional information might be available due to differences in the mechanical properties among whiskers, even a single whisker can transmit large amounts of texture-specific information to its central neural circuits. This occurs because of the match between the feature of the whisker output signal that best distinguishes one texture from another (the kinetic signature of the vibration) and the tuning properties of first-order neurons. Cortical neurons conserve this kinetic signature in their firing patterns.

## Materials and Methods

### 

#### Recording the texture library and construction of the stimulus set

Experiments were conducted in accordance with NIH and institutional standards for the care and use of animals in research. Subjects were ten adult male 250–350-g Wistar rats. In one set of anesthetized rats (urethane, 1.5 g/kg), “electrical whisking” [17,18] was generated by stimulating the right facial nerve (see Figure 1A) with 1–2-V pulses of 100 μsec at 200 Hz for 60 ms to produce whisker protraction, followed by a passive 65-ms whisker retraction. For a selected whisker, horizontal and vertical movements at the base were registered by a two-channel optical sensor, each channel consisting of an LED light source and phototransistor (Video S1). The two voltage signals were digitized (7,634 samples per second). Whisker movement was studied for 10 min under each of six conditions (see Figure 1B). The angle traversed at the whisker base, averaged across all trials and all textures, was 25 degrees (SD ± 1 degree). Average translation across the edge of the textured surface was 3.08 mm (SD ± 0.14 mm).

For each texture, a 50-s continuous record was extracted and sliced into 100 trials of 500 ms, each trial composed of two-dimensional position signals across four 125-ms whisks. For free whisks, a 250-s record was sliced into 500 unique trials. The stimulus set was constructed by splicing trials together at the point of maximum retraction (V_H_ = 0), avoiding the introduction of any position or velocity discontinuity. A free whisk trial always separated two successive texture trials. A block was composed of five different texture trials (t_1–5_) with free whisk trials (fw) interspersed, e.g., fw-t_3_-fw-t_5_-fw-t_1_-fw-t_2_-fw-t_4_. Before stimulus delivery, signals were low-pass filtered at 500 Hz. All data were manipulated in MATLAB software (http://www.mathworks.com).

#### Analysis of vibration kinetics

We sought to quantify the kinetic features that characterized each texture-induced vibration. We refer to the whisker trajectory in one dimension as *x(t)* and the whisker velocity profile as



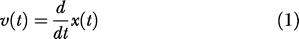



For each texture, we computed the spectrogram |*V(ω,τ_n_)*| of the velocity profile



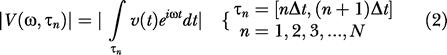



where *Δt* is the 6-ms interval within which each spectrum was computed and *τ_n_* represents the series of *N* sequential time windows. Thus, each spectrogram (see Figure 1, “velocity spectrogram” column) was composed of *N* = 906 spectra.

Considering the duality between the direct and inverse Fourier space



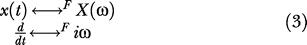



we can substitute Equation 1 into Equation 2, to rewrite Equation 2 as



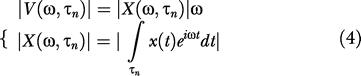



Note that *|X*(*ω,τ_n_*)| also corresponds to the spectrogram of the trajectory in position.

Previous studies have shown that, when the stimulus set consists of sinusoidal whisker movements, the spike count per stimulus for cortical neurons is proportional to the product of the sinusoid's amplitude and frequency, *Xω* [11,12]. To test whether this coding principle extends to natural stimuli, we generalized the measure of *Xω* to the texture-induced vibration. *X* becomes a time-varying spectrum *X(ω,t)*



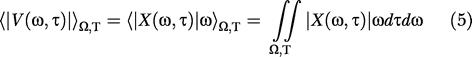



where *T* is the entire time domain and *Ω* is the entire domain of frequency ω.

This quantity—known as the “equivalent noise level”—represents the average amplitude of white noise velocity that dissipates the same average power as the signal of interest. It serves to characterize the entire kinetic signature by a single quantity, equivalent to mean value of the product *Xω* across all time intervals and all values of *ω*.

Our experiments measured velocity in two dimensions, V_H_ and V_V_. Equations 1–5 were generalized to a second dimension by adding, for each time window *τ_n_*, the separately measured spectrograms for the two dimensions. The “velocity spectrogram” column in Figure 1B gives two-dimensional spectrograms as above. Similarly, in Figure 4 the equivalent noise level was calculated in two dimensions, averaged across 100 trials of each texture vibration, and then plotted in relation to neuronal spike count.

#### Measurement of neuronal responses

In a second set of urethane-anesthetized rats, neuronal recordings were made simultaneously from two sites. First-order neurons were recorded by advancing a single electrode (FHC, Bowdoinham, Maine, United States; http://www.fh-co.com) into the right trigeminal ganglion according to stereotaxic coordinates. Cortical recordings were obtained by inserting a 100 microelectrode array (Cyberkinetics, Foxborough, Massachusetts, United States; http://www.cyberkineticsinc.com) to a depth of 700–1,000 μm in the left barrel cortex [35,36]. Ganglion recordings were always single units, whereas cortical recordings consisted of a multiunit cluster at each channel.

The principal whiskers (receptive field centers) in the first-order-only recordings were C3, E1, D6, E6, and γ (twice). The principal whiskers in the cortex-only recordings were A1, B4, E5, and E3 (twice). The principal whiskers in the paired first-order neuron-cortex recordings were δ (twice), E3 (twice), C2 (twice), and E4.

Texture stimuli were delivered to a single whisker using a motor constructed from two orthogonal pairs of parallel piezoelectric wafers driven independently by horizontal and vertical signals (Video S2). The whisker was inserted into a metal tube (0.33 mm inner diameter) with opening 1 mm from the skin. By optically monitoring the whisker shaft, we verified that movements precisely reproduced the previously recorded signals (Figure S1). The second set of rats thus received whisker vibrations identical to those previously recorded during active whisking in the first set of rats.

#### Construction of neuronal tuning curves and response simulations

We selected V_H_ and V_V_ independently from a Gaussian distribution 7,634 times per second. The stimulus was then low-pass filtered (Chebyshev type II) at 500 Hz so as to not exceed the physical capacities of the piezoelectric wafer stimulator (instantaneous reversals of velocity cannot be achieved by any device). The noise stimulus was presented for 10 min after conclusion of the texture stimuli.

We adopted a method of “forward correlation” between stimulus and response where, for all occurrences of a particular stimulus event, the ensuing neuronal spike trains were averaged to construct a response probability profile for that event. This required subdividing velocity space into discrete segments. In Figure 10A, velocity space was partitioned into eight 45-degree angular sectors (*A_1–8_*) and ten radial sectors (*R_1–10_*). At each time point, angle corresponds to the direction of whisker movement, while radial distance corresponds to instantaneous speed. The positions of the radial boundaries were chosen to make each segment contain an equal number of instantaneous velocity events: Because velocity had a Gaussian distribution with a mean of zero, high-velocity events were less common, and consequently the radial boundaries were increasingly widely spaced as distance from zero increased.

To test whether the complex, texture-induced spike patterns followed directly from the tuning curves, a more elaborate analysis was necessary. Each instantaneous velocity (*A,R*) during the whisk gave an ensuing spike probability profile. To simulate a spike train, a spike was generated in each time bin *t* (size 0.13 ms) with probability *P*(*t*), given by the average of the overlying spike probabilities associated with velocity events in the time window before *t* (time window was 1–2 ms before *t* for first-order neurons and 5–20 ms before *t* for cortical neurons). After completion of the simulation, *P*(*t*) was normalized so that the overall simulated spike count matched that in the real data; this normalization did not affect the temporal profile of the simulated PSTH.

## Supporting Information

Figure S1 Comparison of Recorded and Played Back Whisking Trajectories The whisker movements presented as sensory stimuli were accurate reproductions of the movements recorded during electrical whisking in other rats.
(A) Trajectories of two whisks (only horizontal channel shown) recorded during contact with sandpaper P280.
(B) To test playback, a whisker was inserted in the piezoelectric motor guide tube and whisker displacements were measured by the optical sensor placed adjacent to the insertion point of the whisker. The trace shows recordings of the playback of the same two whisks of part A.
(C) Magnified view of the traces indicated in the rectangle in (A) and (B).
(161 KB PDF).

Video S1 Two-Dimensional Optic Sensor and Electrical Whisking Each optic sensor channel consisted of a pair of light tubes (one is a light source, the other leads to a photodiode). The two channels were mounted normal to each other in a metallic ring. Electrical stimulation of cranial nerve VII with 1-V pulses of 100 μs at 200 Hz for 60 ms produced whisker protraction that was followed by a passive 65-ms whisker retraction. The film shows four 125-ms free whisks in the air (8-Hz whisking), consisting of protraction (right to left) and retraction (left to right). Recorded at 500 frames per second with a Motion Scope 500 digital camera (Redlake, San Diego, California, United States; http://www.redlake.com). Rat EW3, whisker C3.
(9.1 MB MOV).

Video S2 Texture Playback with the Two-Dimensional Piezoelectric Motor The motor consists of two pairs of piezoelectric wafers with axes meeting at a mobile joint. The whisker to be stimulated was placed inside the metal tube, which is orthogonal to the joint. The film shows playback of four 125-ms free whisks in the air, consisting of protraction (left to right) and retraction (right to left). Recorded at 500 frames per second.
(9.0 MB MOV).

## Abbreviations

*A_[number]_*: angular sector [number]
fw: free whisk trial
ms: millisecond
PSTH: peristimulus time histogram
*R[number]*: radial sector [number]
SD: standard deviation
t_[number]_: texture trial [number]
V_H_: horizontal velocity
V_V_: vertical velocity
μm: micrometer
