# Supplementary figures and images for: Neuronal Encoding of Texture in the Whisker Sensory Pathway
Source: PLoS Biol. 2005 Jan 11;3(1):e17. doi: 10.1371/journal.pbio.0030017 (PMC544542; doi:10.1371/journal.pbio.0030017)

**A**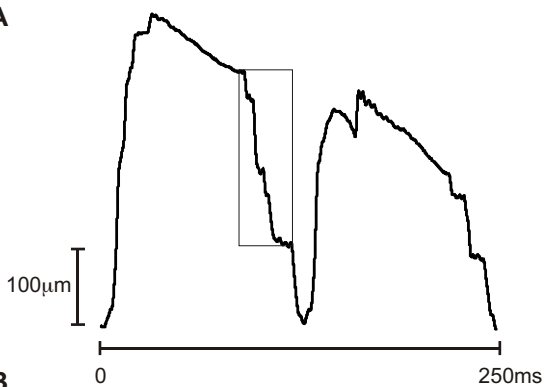**B**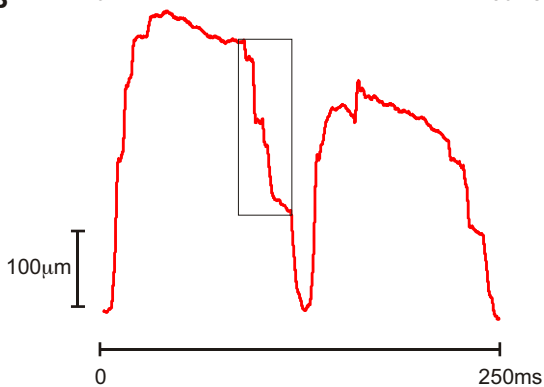**C**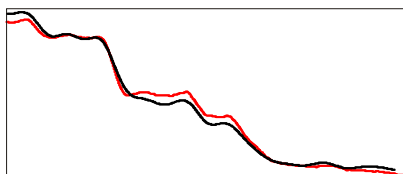

Supplement: Figure S1 — The whisker movements presented as sensory stimuli were accurate reproductions of the movements recorded during electrical whisking in other rats. (A) Trajectories of two whisks (only horizontal channel shown) recorded during contact with sandpaper P280. (B) To test playback, a whisker was inserted in the piezoelectric motor guide tube and whisker displacements were measured by the optical sensor placed adjacent to the insertion point of the whisker. The trace shows recordings of the playback of the same two whisks of part A. (C) Magnified view of the traces indicated in the rectangle in (A) and (B). (161 KB PDF). [file pbio.0030017.sg001.pdf]
